# Level of agreement between 2002 American–European Consensus Group and 2012 American College of Rheumatology classification criteria for Sjögren’s syndrome and reasons for discrepancies

**DOI:** 10.1186/ar4514

**Published:** 2014-03-19

**Authors:** Divi Cornec, Alain Saraux, Béatrice Cochener, Jacques-Olivier Pers, Sandrine Jousse-Joulin, Yves Renaudineau, Thierry Marhadour, Valérie Devauchelle-Pensec

**Affiliations:** 1 CedexService de Rhumatologie, CHRU Brest, Hôpital de la Cavale Blanche, Boulevard Tanguy Prigent, 29609 Brest Cedex, France; 2 EA 2216 Immunologie et Pathologie, SFR ScinBios, Labex ‘Immunotherapy, Graft, Oncology’, Faculté de médecine de Brest, avenue Camille Desmoulins, 29200 Brest, France; 3 Service d'Ophtalmologie, CHRU Brest, Hôpital Morvan, Avenue Foch, 29609 Brest Cedex, France; 4 Service d'Odontologie, CHRU Brest, Hôpital Morvan, Avenue Foch, 29609 Brest Cedex, France; 5 Laboratoire d'Immunologie, CHRU Brest, Hôpital Morvan, Avenue Foch, 29609 Brest Cedex, France; 6 Service de Rhumatologie, Hôpital de la Cavale Blanche, BP 824, F 29609 Brest Cedex, France

## Abstract

**Introduction:**

The aims of this study were to assess agreement between the currently used 2002 American–European Consensus Group (AECG) classification criteria and the new 2012 American College of Rheumatology (ACR) criteria for Sjögren’s syndrome (SS) and to identify potential sources of disagreement.

**Methods:**

We studied 105 patients between 2006 and 2013 from the Brittany cohort of patients with suspected SS. AECG criteria were applied using only Schimer’s test and unstimulated whole salivary flow (UWSF) to assess objective ocular and oral involvement, since these are the tests most physicians use in clinical practice. Agreement between the two sets of criteria was assessed using Cohen’s κ coefficient.

**Results:**

Of those studied, 42 patients fulfilled AECG and 35 ACR criteria. Agreement between the two sets was moderate (κ = 0.53). Patients fulfilling ACR but not AECG criteria (n = 8) were significantly younger and had shorter symptom durations, but only three of them had SS in the opinion of the evaluating physician. Xerostomia and xerophthalmia (AECG set only) did not discriminate between patients with and without SS. The use of UWSF in the AECG but not the ACR criteria explained part of the disagreement. The serological item in the ACR set (positive rheumatoid factor and antinuclear antibody ≥1:320 or anti-SSA/SSB positivity) did not result in classification differences compared to anti-SSA/SSB antibody alone (AECG set). Agreement between ocular staining score ≥3 (ACR set) and Schirmer’s test ≤5 mm/5 min (AECG set) was very low (κ = 0.14).

**Conclusions:**

Agreement was only moderate between ACR and AECG criteria, suggesting these two sets would not select comparable patient populations. An international consensus about which classification criteria should be used in clinical studies is needed.

## Introduction

The classification criteria for Sjögren’s syndrome (SS) issued in 2002 by the American–European Consensus Group (AECG) have been widely used in clinical studies over the last decade [1]. In 2012, the Sjögren’s International Collaborative Clinical Alliance issued new classification criteria, which have been endorsed by the American College of Rheumatology (ACR) [2]. These new criteria are intended for use in patients referred to specialists because of signs or symptoms suggesting SS. They were developed by asking experts in rheumatology, ophthalmology and oral medicine to select the items they felt were most relevant.

The new criteria differ substantially from the 2002 AECG criteria in three ways: they include no subjective ocular and oral symptoms and no functional or morphological tests for the salivary glands; they use a new ocular staining score (OSS) [3] as the only criterion for ocular involvement; and they allow the use of an antinuclear antibody (ANA) titer ≥1:320 plus rheumatoid factor (RF) positivity as an alternative to anti-SSA/SSB antibody positivity for the assessment of systemic autoimmunity.

Here, our objectives were to evaluate agreement between the two criteria sets and to identify sources of disagreement.

## Methods

### Study population

We studied the single-centre Brittany cohort of patients with suspected SS included between November 2006 and March 2013 in Brittany, France. The inclusion criteria are described elsewhere [4]. Briefly, patients were addressed to our consultation by their family physician, rheumatologist, internist, dentist or ophthalmologist if SS was suspected due to sicca complaints, major salivary gland swelling, suggestive extraglandular features or suggestive autoantibodies. Written consent was obtained from all participants, and the study was approved by the local ethics committee (Brest University Hospital).

This study included 105 patients of the cohort who had all the tests available to apply both ACR and AECG criteria, including 99 (94.3%) women. Mean age was 57.2 ± 13.7 years and mean symptom duration was 6.7 ± 6.1 years.

### Clinical evaluation and laboratory tests

Schirmer’s test was considered abnormal if ≤5 mm/5 minute and the unstimulated whole salivary flow if <0.1 ml/minute. ANAs were assessed on HEp-2 cells and anti-SSA and anti-SSB antibodies using commercial enzyme-linked immunosorbent assays, and RF (IgM and IgA isotypes) using in-house enzyme-linked immunosorbent assays. Minor labial salivary gland biopsy was performed in all patients and graded according to the semi-quantitative score of Chisholm and Mason [5]. Salivary gland biopsy grades 3 and 4 indicating focus scores ≥1/4 mm^2^ were considered abnormal.

### Ophthalmologic evaluation

All patients underwent a slit-lamp examination by an ophthalmologist experienced in dry-eye diseases. The corneal fluorescein pattern was graded from 0 (no punctate epithelial erosions) to 3 (severe keratitis). A drop of lissamine green dye was then instilled into the inferior conjunctival fornix of each eye, the patient was asked to blink several times, and the nasal and temporal bulbar conjunctivae were then immediately graded semi-quantitatively from 0 (no staining) to 3 (diffuse dot staining or confluent staining).

The Sjögren’s International Collaborative Clinical Alliance OSS [3] was published during the inclusion period for our cohort. We retrospectively computed the OSS of both eyes as the 0 to 12 sum of the 0 to 3 lissamine green scores for the nasal and temporal conjunctiva (range, 0 to 6) plus the 0 to 3 fluorescein score for each cornea multiplied by 2 (because the specific patterns of corneal fluorescein staining giving additional points in the OSS (central staining, filaments or confluent staining) were not described in our protocol). OSS ≥3 in at least one eye was considered abnormal.

### Case ascertainment

Table 1 presents the rules for classification using the AECG and ACR criteria sets. AECG criteria were applied using only Schirmer’s test and unstimulated whole salivary flow, which are the tests we use in clinical practice to assess objective ocular and salivary gland involvement.

**Table 1:** **Pragmatic AECG**[1]**and ACR**[2]**classification criteria for Sjögren’s syndrome**

	**Pragmatic 2002 AECG criteria**	**2012 ACR criteria**
Items	1. Ocular dryness symptoms	1. Positive anti-SSA or anti-SSB antibodies or positive rheumatoid factor plus ANA ≥1:320
	2. Oral dryness symptoms	2. Focus score ≥1 focus/4 mm^2^ on minor salivary gland biopsy
	3. Ocular signs: Schirmer’s test ≤5 mm/5 minutes	3. Keratoconjunctivitis sicca with ocular staining score ≥3
	4. Focus score ≥1 focus/4 mm^2^ on minor salivary gland biopsy	
	5. Salivary gland involvement: unstimulated whole salivary flow ≤0.1 ml/minute	
	6. Positive anti-SSA or anti-SSB antibodies	
Rules for classification	Presence of any four of the six items with at least item 4 or 6, or presence of any three of the four objective items (items 3, 4, 5 and 6)	In a patient with suspected Sjögren’s syndrome, any two of the three items

Exclusion criteria for both criteria sets are head-and-neck radiation, graft-versus-host disease, hepatitis C infection, acquired immunodeficiency syndrome or sarcoidosis. Pre-existing lymphoma and use of anticholinergic drugs are exclusion criteria only in the AECG criteria, whereas amyloidosis and IgG4-related disease are exclusion criteria only in the ACR criteria.

We did not use in this study the whole set of AECG criteria, but only the tests we use in clinical practice (pragmatic AECG criteria); that is, only the unstimulated whole salivary flow to assess salivary gland involvement (and not salivary scintigraphy or parotid sialography which are considered obsolete by most physicians), and only the Schirmer’s test to assess ocular signs (and not vital dye staining graded according to van Bijsterveld method). ACR, American College of Rheumatology; AECG, American–European Consensus Group.

The evaluating physician was also asked to define the most probable diagnosis in his opinion for each patient, without referring to specific classification criteria. All cases were reviewed by a panel of three experts (VD-P, AS and SJ-J) to reach consensus. The most probable diagnoses in the opinion of the experts were SS in 47 (44.8%) patients, idiopathic sicca syndrome in 37 (35.2%) patients, other connective tissue diseases in 11 (10.5%) patients and drug-induced sicca syndrome in 10 (9.5%) patients.

This clinical definition of SS cases was not used as a gold standard to compare the diagnostic performance of AECG criteria and ACR criteria in terms of sensitivity and specificity, since the physician most probably used, even subconsciously, current validated classification criteria (that is, AECG criteria) to perform his diagnosis, leading to circular reasoning that would have overestimated AECG performance to the detriment of ACR criteria.

### Statistical analysis

Statistical tests were performed using the Statistical Package for the Social Sciences (SPSS 18.0, 2009; SPSS Inc., Chicago, IL, USA). Quantitative variables were described as mean ± standard deviation and qualitative variables as number (%). Agreement between classification criteria sets and between criteria was evaluated using Cohen’s kappa coefficient (κ). To compare patient groups, we used the Mann–Whitney test, Fisher’s exact test or the chi-square test as appropriate.

## Results

### Classification criteria

Of the 105 patients, 42 (40.0%) fulfilled the AECG criteria and 35 (33.3%) fulfilled the ACR criteria (Table 2). Agreement between the two criteria sets was moderate (κ = 0.53). Patients fulfilling only the ACR criteria were significantly younger and had shorter symptom durations than did patients fulfilling the AECG criteria (mean age, 46.6 ± 15.8 vs. 60.0 ± 11.4 years; and mean symptom duration, 2.9 ± 2.4 vs. 7.7 ± 6.8 years; *P* < 0.001 for both comparisons).

**Table 2:** Characteristics of patients meeting ACR and/or AECG criteria for Sjögren’s syndrome

	**Patients fulfilling**			
	**Both sets**	**ACR set only**	**AECG set only**	**Neither set**
	**(*n*** **= 27)**	**(*n*** **= 8)**	**(*n*** **= 15)**	**(*n*** **= 55)**
Age (years)	60.7 ± 11.7	46.6 ± 15.8	59.3 ± 11.1	56.4 ± 14.5
Symptom duration (years)	9.5 ± 7.4	2.9 ± 2.4	4.7 ± 4.3	6.4 ± 5.7
Female	25 (92.6)	8 (100)	14 (93.3)	52 (94.5)
Xerophthalmia	26 (96.3)	6 (75.0)	14 (93.3)	50 (90.9)
Xerostomia	27 (100)	6 (75.0)	15 (100)	51 (92.7)
Salivary flow ≤0.1 ml/minute	18 (66.7)	0 (0)	10 (66.7)	16 (29.1)
Schirmer’s test ≤5 mm/5 minutes	22 (81.5)	1 (12.5)	12 (80.0)	13 (23.6)
OSS ≥3	24 (88.9)	6 (75.0)	0 (0)	19 (34.5)
Anti-SSA or anti-SSB positivity	18 (66.7)	4 (50.0)	4 (26.7)	1 (1.8)
ANA ≥1:320	25 (92.6)	5 (62.5)	8 (53.3)	18 (32.7)
RF positivity	14 (51.9)	4 (50.0)	1 (6.7)	6 (10.9)
Anti-SSA/SSB or RF plus ANA ≥1:320	21 (77.8)	4 (50.0)	4 (26.7)	1 (1.8)
Focus score ≥1	24 (88.9)	7 (87.5)	11 (73.3)	6 (10.9)

Data presented as mean ± standard deviation or number (%). Xerophthalmia and xerostomia referred to subjective complaints from the patients. ACR, American College of Rheumatology; AECG, American–European Consensus Group; OSS, ocular staining score; ANA, antinuclear antibody; RF, rheumatoid factor.

### Description of the patients with discordant classification

Table 3 details the features of the eight patients fulfilling ACR criteria but not AECG criteria and of the 15 patients fulfilling AECG criteria but not ACR criteria. All patients fulfilling only AECG criteria had sicca complaints, either abnormal unstimulated whole salivary flow or Schirmer’s test, and either anti-SSA/SSB antibodies or abnormal salivary gland biopsy. Five of them had extraglandular involvement, and all of them had SS in the opinion of the evaluating physician.

**Table 3:** Description of the patients fulfilling only ACR criteria or only AECG criteria

**Patient**	**Sex**	**Age (years)**	**Duration (years)**^**a**^	**Eye**^**b**^	**Mouth**^**c**^	**Arthritis**^**d**^	**Lung**^**e**^	**Neuro**^**f**^	**Parotid**^**g**^	**UWSF**^**h**^	**Schirmer**^**i**^	**OSS**^**j**^	**Chisholm**^**k**^	**ANA**	**Anti-SSA**^**l**^	**Anti-SSB**^**l**^	**RF (*n*** **< 0.2)**	**ACPA (*n*** **< 20)**	**IgG (mg/l)**	**Clinical**^**m**^
**Detailed characteristics of the eight patients fulfilling only ACR criteria**																				
1	F	25	0	1	0	0	0	0	0	0.35	8	3	1	160	146	46	0.14	9	9.2	SS
2	F	63	1	1	1	0	0	0	0	0.4	30	3	4	1,280	0	0	0	0	5.8	SS
3	F	34	1	1	0	1	0	0	0	0.2	20	4	4	1,280	174	0	0.5	250	16.2	RA
4	F	59	5	0	1	0	0	0	1	0.4	10	1	3	640	140	80	0.9	7	13.4	SS
5	F	61	5	1	1	0	0	0	0	0.5	8	3	3	0	0	0	0	0	12.1	Sicca
6	F	47	4	1	1	0	0	0	0	0.2	30	3	3	1,280	0	0	0	0	16.5	UCTD
7	F	57	1	1	1	1	0	1	0	0.5	30	4	4	160	0	0	0.52	250	12.7	RA
8	F	27	6	0	1	0	1	0	0	0.25	30	0	3	1,280	100	0	0.81	15	18.9	SLE
**Detailed characteristics of the 15 patients fulfilling only AECG criteria**																				
1	F	67	1	1	1	0	0	0	0	0.005	5	2	3	160	0	0	0	0	11.7	SS
2	F	45	1	1	1	0	0	0	0	0.001	5	2	2	640	154	0	0.05	0	13	SS
3	F	75	0	1	1	0	0	0	0	0.2	0	2	3	0	0	0	0.34	0	9.3	SS
4	F	56	1	1	1	0	0	0	0	0.005	25	2	2	1,280	24	152	0	0	9.9	SS
5	F	50	13	1	1	0	0	1	1	0.1	20	0	4	160	0	0	0	0	9.1	SS
6	F	69	6	1	1	0	0	0	0	0.25	5	1	4	1,280	0	0	0	0	10.3	SS
7	F	66	1	1	1	1	0	0	0	0.486	5	2	3	320	0	0	0	0	7	SS
8	F	55	7	1	1	0	1	0	0	0.02	15	2	4	160	0	0	0	0	7.1	SS
9	F	43	3	1	1	0	0	0	0	0.15	5	1	1	640	85	0	0	0	12.6	SS
10	F	78	10	1	1	0	0	0	0	0.05	7	2	3	160	0	0	0	0	9.7	SS
11	M	64	2	1	1	0	1	0	0	0.2	0	0	4	320	0	0	0	28	22.6	SS
12	F	60	8	1	1	0	0	1	1	0.05	5	2	3	640	9	32	0	72	8.43	SS
13	F	58	2	1	1	0	0	0	0	0.04	4	0	3	0	0	0	0	0	8.33	SS
14	F	43	10	0	1	0	0	0	0	0.06	0	1	2	320	40	0	0	0	10	SS
15	F	50	1	1	1	0	0	0	0	0.05	15	1	3	0	0	0	0	0	9.7	SS

ACPA, anti-citrullinated peptide antibodies; ACR, American College of Rheumatology; AECG, American–European Consensus Group; ANA, antinuclear antibody; F, female; IgG, serum level of immunoglobulin G; M, male; OSS, ocular staining score; SS, Sjögren’s syndrome. RA, rheumatoid arthritis; RF, rheumatoid factor; sicca, idiopathic sicca syndrome; SLE, systemic lupus erythematosus; UCTD, undifferentiated connective tissue disease; UWSF, unstimulated whole salivary flow. ^a^Duration of the symptoms. ^b^Xerophthalmia (0, absent; 1, present). ^c^Xerostomia (0, absent; 1, present). ^d^Presence of swollen and tender joints on the day of examination. ^e^Includes the presence of chronic dry cough or computed tomography-proven interstitial lung disease. ^f^Patient with objective peripheral neuropathy. ^g^Presence or history of parotidomegaly. ^h^Abnormal if ≤0.1 ml/minute. ^i^Schirmer’s test abnormal if ≤ 5 mm/5 minutes. ^j^Abnormal if ≥ 3. ^k^Chisholm’s grading of the lymphocytic infiltration on minor salivary gland biopsy (grades 3 and 4 correspond to a focus score ≥1 focus of more than 50 lymphocytes/4 mm^2^). ^l^Anti-SSA and anti-SSB antibodies (*n* < 40). ^m^Diagnosis made by the physician, without reference to specific classification criteria.

Among the eight patients fulfilling only ACR criteria, none of them had abnormal unstimulated whole salivary flow or Schirmer’s test, but seven had abnormal salivary gland biopsy. Only three of them had SS according to the physician’s opinion, and other more likely diagnoses were rheumatoid arthritis (*n* = 2), systemic lupus erythematosus (*n* = 1), undifferentiated connective tissue disease (*n* = 1) and idiopathic sicca syndrome (*n* = 1).

### Subjective sicca complaints (AECG set only)

The subjective sicca symptoms (xerophthalmia and xerostomia) were noted in nearly all of the patients (respectively in 92.4% and 94.3%), suggesting no ability of these symptoms to discriminate between patients with and without SS in this population. The proportions of patients with xerophthalmia or xerostomia were not significantly different in the group fulfilling both criteria sets and in the group fulfilling neither criteria set (*P* = 0.35 and *P* = 0.2, respectively).

### Functional salivary-gland assessment (AECG set only)

Salivary flow was decreased in 66.7% of patients fulfilling both criteria sets and in 29.4% of those fulfilling neither criteria set (*P* = 0.001). No patient fulfilling only the ACR set had a decrease in salivary flow.

### Serological criterion

Only three patients had positive RF plus ANA ≥ 1:320 but negative anti-SSA antibodies; that is, met the ACR serological criterion but not the AECG serological criterion. These three patients fulfilled the AECG criteria; they also fulfilled the ACR criteria even without taking RF and ANA into account, since they all had abnormal OSS and focus score results.

### Ophthalmological criterion

Agreement between the OSS (ACR set) and Schirmer’s test (AECG set) was very low (κ = 0.14). Agreement with the salivary gland biopsy was lower for the OSS than for Schirmer’s test (κ = 0.14 vs. 0.35, respectively). Both the OSS and Schirmer’s test showed poor agreement with anti-SSA/SSB positivity (κ = 0.21 and κ = 0.27 respectively).

## Discussion

In this study, agreement was only moderate between the AECG and ACR criteria sets. The AECG criteria set classified more patients as having SS, whereas the ACR criteria set seemed to classify patients earlier in the course of the disease but included patients who did not have SS in the opinion of the physician. These discrepancies were chiefly ascribable to the absence in the ACR set of functional salivary gland testing (such as salivary flow measurement) and, above all, to the intrinsic differences between the OSS and Schirmer’s test.

These two ocular dryness tests had very low agreement. According to the latent class analysis method used to develop the ACR classification criteria [2], the OSS had 89.7% sensitivity but only 37.8% specificity for SS, whereas Schirmer’s test had a lower sensitivity of 42.7% but a better specificity of 75.1%. In our study, in patients fulfilling neither criteria set (and who were therefore unlikely to be diagnosed with SS), an abnormal OSS was more common than an abnormal Schirmer’s test, suggesting lower specificity of the OSS. To compare the diagnostic usefulness of these two tests, we did not use the physician’s diagnosis as the reference, since this diagnosis relied chiefly on Schirmer’s test and not on the OSS. Instead, we evaluated the agreement of the ocular dryness tests with the focus score and anti-SSA/SSB positivity, two major diagnostic features of SS [6] that served as external validation criteria. Compared with the OSS, Schirmer’s test showed slightly better agreement with the focus score.

In the recently published study by Rasmussen and colleagues, the discordance between AECG and ACR criteria was also mostly attributed to the differences between the tests assessing the objective ocular component [7]. In their study, OSS displayed a poor specificity for SS (45 to 51%), which could be partially corrected by increasing its positivity cutoff value from ≥ 3 to ≥ 4/12.

A complete ophthalmological evaluation is mandatory in patients with suspected SS, in particular to assess eyelid diseases and the differential diagnoses of keratoconjunctivitis sicca. However, classification tools should rely on the most specific items. Advantages of Schirmer’s test include ease of use, even at the bedside in any clinical ward where trained staff members are available, and good performance as a screening tool for SS [8].

The serological item of the AECG criteria could probably be improved, since roughly 40% of primary SS patients do not have anti-SSA/SSB antibodies. However, we have shown here that the adjunction of ANA and RF, as proposed in the ACR criteria, did not modify the classification potential of anti-SSA/SSB alone. Other tests should be evaluated when new classification criteria will be developed, such as blood B-cell phenotyping [9] or other autoantibodies [10].

Another intrinsic difference between the AECG criteria and the ACR criteria is the absence of items assessing the subjective component of the disease in the latter. Indeed, the ACR criteria do not target the general population but only patients with suspected SS, who complain most of the time of sicca symptoms as in our study. Conversely, the AECG criteria may be applied to any patient thanks to the inclusion of the symptoms in items 1 and 2 (see Table 1). However, the preliminary European criteria and then the AECG criteria were not designed to be used in the general population, since the control patients enrolled in their development study ‘were to be selected from those subjects referred to an SS expert because of ocular or oral signs and symptoms that simulated the clinical manifestations of SS, and for whom a complete evaluation was justified in order to establish a differential diagnosis’ [11]. The precise diagnostic value of several questionnaires assessing ocular and oral symptoms has been carefully evaluated in these patients with suspected SS [12]. Such questionnaires may be valid tools for SS screening in the general population [8], but in our study the subjective symptoms did not participate in the discrepancy between AECG and ACR criteria, since nearly all patients had sicca complaints.

An earlier study compared the AECG criteria, the ACR criteria and the Japanese classification criteria sets for SS [13]. Taking the physician’s diagnosis as the reference standard, the AECG and ACR criteria sets had 78.6% and 77.5% sensitivity, respectively, and 90.4% and 83.5% specificity.

However, a direct comparison of the AECG and ACR criteria sets in terms of sensitivity and specificity, taking the physician’s diagnosis as the reference standard, may be inherently biased, as physicians probably rely heavily on the currently used AECG criteria set to diagnose SS. The resulting circular reasoning may overestimate the metrological features of the AECG criteria set.

Classification criteria for systemic diseases such as SS, rheumatoid arthritis, systemic lupus erythematosus, systemic sclerosis and inflammatory myopathies are designed to improve the homogeneity of populations enrolled in clinical studies, in order to allow valid comparisons across studies [14-17]. Since no specific reference standard is available for diagnosing these complex diseases, classification criteria are often used for diagnostic purposes, despite their limitations [18-20].

Whether classification criteria should have a higher sensitivity or specificity is a matter of debate. Foremost, classification criteria might be used to recruit patients in epidemiological studies; for example, to describe the whole spectrum of a disease including the mildest forms, and to define prognostic factors. For such a study, the classification criteria sensitivity should be high, even to the detriment of their specificity, since it would not be dangerous to include patients for whom the diagnosis is not definitely certain. Conversely, in therapeutic trials using potentially harmful drugs such as immunosuppressant or biological therapies, one could not take the risk of including patients who do not have the disease. In that case, the classification criteria have to be the most specific possible. All published classification criteria for SS, including preliminary European and AECG criteria, have a very high specificity (95 to 100%) but variable sensitivity (60 to 95%) [21]. The scientific community must achieve the widest consensus for a new classification system, which would display the best combination of sensitivity and specificity in order to be used universally in both epidemiological and therapeutic studies. To achieve this goal, a large international study is warranted, and the new classification criteria should include new diagnostic tools that were validated recently, such as salivary gland ultrasonography [4,22-24].

## Conclusions

In this study, agreement was only moderate between the AECG and ACR criteria sets. This discrepancy was mainly ascribable to the intrinsic differences between the tests assessing the ocular component of the criteria. Our results suggest that the ACR criteria may detect early forms of disease affecting specific SS subpopulations such as those with negative anti-SSA/SSB autoantibodies. On the other hand, the AECG criteria seem definitely more specific but also more stringent. The existence of two different classification criteria sets for SS that select different patient populations may cause confusion [25,26]. An international study under the auspices of both the ACR and the European League Against Rheumatism is warranted to develop new universally recognized classification criteria, which would probably include new procedures such as major salivary gland ultrasonography.

## Abbreviations

ACR: American College of Rheumatology; AECG: American–European Consensus Group; ANA: antinuclear antibody; OSS: ocular staining score; RF: rheumatoid factor; SS: Sjögren’s syndrome.

## Competing interests

The authors declare that they have no competing interests.

## Authors’ contributions

DC was responsible for conception and design, data collection, statistical analysis, manuscript writing and final approval of the manuscript. AS was responsible for conception and design, data collection, statistical analysis, critical revision and final approval of the manuscript. BC, J-OP, SJ-J, YR and TM were responsible for data collection and analysis, critical revision and final approval of the manuscript. VD-P was responsible for conception and design, data collection and analysis, manuscript writing and final approval of the manuscript. All authors read and approved the final manuscript.
